# Grand Total EEG Score Can Differentiate Parkinson's Disease From Parkinson-Related Disorders

**DOI:** 10.3389/fneur.2019.00398

**Published:** 2019-04-18

**Authors:** Ela Austria Barcelon, Takahiko Mukaino, Jun Yokoyama, Taira Uehara, Katsuya Ogata, Jun-ichi Kira, Shozo Tobimatsu

**Affiliations:** ^1^Department of Neurology, Neurological Institute, Graduate School of Medical Sciences, Kyushu University, Fukuoka, Japan; ^2^Department of Clinical Neurophysiology, Neurological Institute, Graduate School of Medical Sciences, Kyushu University, Fukuoka, Japan

**Keywords:** idiopathic Parkinson's disease, atypical Parkinsonian disorders, corticobasal degeneration, multiple system atrophy, progressive supranuclear palsy, grand total EEG score

## Abstract

**Background:** Semi-quantitative electroencephalogram (EEG) analysis is easy to perform and has been used to differentiate dementias, as well as idiopathic and vascular Parkinson's disease.

**Purpose:** To study whether a semi-quantitative EEG analysis can aid in distinguishing idiopathic Parkinson's disease (IPD) from atypical parkinsonian disorders (APDs), and furthermore, whether it can help to distinguish between APDs.

**Materials and Methods:** A comprehensive retrospective review of charts was performed to include patients with parkinsonian disorders who had at least one EEG recording available. A modified grand total EEG (GTE) score evaluating the posterior background activity, and diffuse and focal slow wave activities was used in further analyses.

**Results:** We analyzed data from 76 patients with a final diagnosis of either IPD, probable corticobasal degeneration (CBD), multiple system atrophy (MSA), or progressive supra-nuclear palsy (PSP). IPD patients had the lowest mean GTE score, followed those with CBD or MSA, while PSP patients scored the highest. However, none of these differences were statistically significant. A GTE score of ≤9 distinguished IPD patients from those with APD (p < 0.01) with a sensitivity of 100% and a specificity of 33.3%.

**Conclusion:** The modified GTE score can distinguish patients with IPD from those with CBD, PSP or MSA at a cut-off score of 9 with excellent sensitivity but poor specificity. However, this score is not able to distinguish a particular form of APD from other forms of the disorder.

## Introduction

Parkinsonian disorders comprise a heterogenous group of neurodegenerative diseases that manifest the core symptoms of parkinsonism, which include bradykinesia, rigidity, resting tremor, loss of postural reflexes, flexed posture, and freezing of gait (1–3). Idiopathic Parkinson's disease (IPD) is the second most common neurodegenerative disorder worldwide (4) and the most common disease entity in this group of disorders. Atypical parkinsonian disorders (APD), also known as “Parkinson-plus syndromes,” such as progressive supranuclear palsy (PSP), multiple system atrophy (MSA), and corticobasal degeneration (CBD), account for about 10–20% of parkinsonian disorders (5). APDs have more severe symptoms, exhibit more complications, progress more rapidly, and consequently lead to a shorter survival time than IPD (6–8). The differences between these forms of parkinsonian disorders in terms of prognosis and management have led to the recognition that it is important to distinguish between these disease entities. Furthermore, in the field of research, distinguishing between these disorders is vital, as firm conclusions from studies can only be made if the sample populations are as homogenous as possible (2).

Misdiagnosis of IPD, especially in its early stages, can be as high as 20–30%. One of the reasons identified as accounting for misdiagnosis is the failure to recognize APD (9). Approximately 50–70% of CBD patients are never diagnosed by primary neurologists, only 75% of PSP patents are clinically diagnosed by specialists, and only 50% of MSA patients are diagnosed correctly by primary neurologists (10–12). The high misdiagnosis rate is clear evidence that the distinction of IPD from APD remains a challenge to clinicians, despite the advent of new technological advancements in medicine (13, 14).

New and relevant information revealed by novel research allows the different diagnostic criteria for IPD, CBD, MSA, and PSP to be continually revised and updated, paving the way for improved accuracy (13, 15–17). Studies of these diagnostic criteria have emphasized the importance of identifying core symptoms and performing confirmatory laboratory tests. While it is undeniable that novel diagnostic tools such as dopamine transporter (DAT) scans, meta-iodobenzylguanidine (mIBG) scintigraphy, cerebrospinal fluid (CSF) protein analysis, and positron emission tomography (PET) ligand marker imaging provide significant information that greatly improves diagnosis of movement disorders, they are not readily available or easy to perform, especially in developing countries. Electroencephalography (EEG) is a common, non-invasive diagnostic modality that is used to assess functional changes in the brain (18). Many studies have used EEG to investigate cognitive impairment and movement disorders. With the advent of novel technologies, advanced EEG analysis, and in particular quantitative EEG, is gaining attention as a useful method to study these neurological disorders (19, 20). However, for the general clinician and for general neurologists, examination with quantitative EEG is tedious to perform. Moreover, at least for dementia, visual EEG analysis has as much diagnostic value as quantitative EEG (18). The grand total EEG (GTE) score is a form of a semi-quantitative EEG analysis that is relatively easy to perform. It has been proven to be effective in differentiating Alzheimer's Disease from dementia with Lewy bodies (21, 22), and IPD from vascular parkinsonism (23). Therefore, this study aimed to investigate whether a simple semi-quantitative EEG analysis can be helpful in distinguishing IPD from APD and, furthermore, in distinguishing between specific forms of APD. To achieve this, we retrospectively reviewed the EEG findings of parkinsonian patients admitted to Kyushu University Hospital, a tertiary hospital.

## Materials and Methods

### Patients

Charts at the Kyushu University Hospital were reviewed to include data from all patients matching the following criteria: referred to the Department of Neurology from 2006 to 2018; ≥40 years of age; admitted with parkinsonian disorder as the diagnosis, or with parkinsonian symptoms as the chief complaint (i.e., gait disturbance or ataxia, tremor, involuntary movement, or slowness of movement); and with at least one EEG recording. Patients below 40 years of age or with altered sensorium during the EEG recording were excluded from this study.

Patient data were retrieved from charts in the hospital's online database. The differential diagnoses made for each patient were a result of an integrated analysis of all the available clinical information and diagnostic examinations, including: cranial magnetic resonance imaging; single photon emission computed tomography (SPECT); fluorodeoxyglucose positron emission tomography; DAT scan, and mIBG scintigraphy. Final patient diagnoses were decided collaboratively by a team of neurological experts with the aid of updated clinical guidelines (15–17, 24). Only data from patients with a final diagnosis of IPD, PSP, probable CBD (24), and MSA (MSA-P, MSA-C, or unspecified) were included in the analysis. Patients with uncertain diagnoses were excluded, namely those with: suspected or diagnosed diffuse Lewy body disease or probable or possible Parkinson's disease with dementia, as defined by the Movement Disorder Society criteria (25). Experimental procedures for this study were approved by the Ethics Committee at the Graduate School of Medical Sciences, Kyushu University.

### EEG Recording

All patient EEG data were previously recorded using a 19-channel digital EEG recording system (Nihon Kohden, Japan). Electrodes were placed according to the International 10–20 system and included electrodes recording the electrocardiogram and electrooculogram (EOG). EOG was recorded from the electrode at the lower medial canthus, referenced to the right ear (A2). The sampling rate was set at 500 Hz with a bandpass filter of 0.5–60 Hz. Impedances were kept below 10 kΩ. Recordings were performed for at least 30 min while the patient was awake and relaxed, and involved activation methods such as eye opening/closing, intermittent photic stimulation (3–21 Hz in 3-Hz increments) and hyperventilation, unless contraindicated.

### EEG Assessment

EEG recordings were visually assessed at a sensitivity of 10 μV, a time constant of 0.3 s, and a time window of 10 s per epoch. Each EEG recording was reviewed by a neurologist (EB), the first reviewer, and by a board-certified neurophysiologist (ST), the second reviewer. The GTE Score was evaluated for each patient (Table 1). We adopted the GTE score, but modified the original version used in previous studies (21, 22), to assess the EEG findings more precisely. More specifically, we expanded the assessment of background activity, taking into account the distribution, amplitude, and symmetry of the EEG. In addition to this, we also gave importance to the prevalence and the reactivity of diffuse and focal slow wave activities. Consensus scoring by the two reviewers (EB and ST) on each GTE subcomponent, as well as on the total GTE score was used to rate each EEG recording, with both reviewers blind to the final diagnosis during scoring. Data from the EOG channels were also examined and abnormalities were noted.

**Table 1:** Modified grand total electroencephalogram score.

**MODIFIED GRAND TOTAL EEG SCORE**		
Frequency of rhythmic background activity	None	0
	8–9 Hz	1
	7–8 Hz	2
	6–7 Hz	3
	4–6 Hz	4
Distribution of rhythmic background activity	Occipito-temporal-parietal	0
	Up to central	1
	Up to frontal	2
Background activity amplitude	Low to high voltage (>20 μV in bipolar montage)	0
	Very low voltage (<20 μV in bipolar montage)	1
Reactivity of rhythmic background activity	Normal	0
	Decreased with eye opening	1
	Absent with eye opening	2
	Absent with auditory stimulus	3
	Absent with pain stimulus	4
Asymmetry of rhythmic background activity	Symmetric	0
	Asymmetric	1
Photic driving response	Absent	−
	Present	+
Diffuse slow wave activity	None	0
	Intermittent theta	1
	Intermittent theta + sporadic delta	2
	Intermittent theta + intermittent delta	3
Reactivity of diffuse slow wave activity	Decreased with eye opening	0
	Decreased with auditory but not to eye opening	1
	Decreased with pain stimulus only	2
	No reactivity	3
Prevalence of diffuse slow activity	None	0
	Rare <1%	1
	Occasional 1–9%	2
	Frequent 10–49%	3
	Abundant 50–89%	4
	Continuous >90%	5
Paroxysmal activity	None	0
	Paroxysmal slow wave activity	3
	Frontal Intermittent Delta Activity (FIRDA)	5
Focal slow wave activity	None	0
	Intermittent theta	1
	Intermittent theta + sporadic delta	2
	Intermittent theta + intermittent delta	3
Reactivity of focal slow wave activity	Decreased with eye opening	0
	Decreased with auditory; not to eye opening	1
	Decreased with pain stimulus only	2
	No reactivity	3
Prevalence of focal slow activity	None	0
	Rare <1%	1
	Occasional 1–9%	2
	Frequent 10–49%	3
	Abundant 50–89%	4
	Continuous >90%	5
Location of focal slow wave	None	0
	Moderate unilateral ± mild contralateral	1
	Moderate bilateral	2
	Severe unilateral moderate contralateral	3
	Severe bilateral	4
	Multifocal	5
Epileptic focal abnormalities—location	None	0
	Moderate unilateral	1
	Moderate bilateral	2
	Severe unilateral moderate contralateral	3
	Severe bilateral	4
	Multifocal	5
Epileptic focal abnormalities—type	None	0
	Sporadic sharp waves	2
	Frequent sharp waves	3
	Triphasic waves	4
	Periodic Lateralized Epileptiform Discharges (PLEDs)	5

*The minimum possible score is 0 and the maximum possible score is 54*.

### Statistical Analyses

SPSS 24.0 was used to perform statistical analyses. Analysis of variance (ANOVA), and chi-square tests were performed to compare the sex and mean age of the sample population, respectively. Chi-square test or Fisher's exact test were used to compare proportions of frequencies. ANOVA was used to compare the mean GTE score and the means of its subcomponents between the four subgroups of patients with differing diagnoses. Receiver operating characteristic (ROC) curves were fit by plotting the true-positive rate (sensitivity) against the false-positive rate (100—specificity). The cut-off point for differentiating IPD from APD with optimal sensitivity and specificity from the total GTE score was identified using the ROC curves. Possible interaction effects of GTE score with cognitive scores such as the Mini-Mental Status Examination (MMSE), the Frontal Assessment Battery (FAB), and with patient age were investigated by performing an analysis of covariance (ANCOVA).

## Results

### Patient and EEG Record Selection Process

A total of 627 medical records were retrospectively reviewed, 347 of which had an initial diagnosis falling under the category of parkinsonian disorder or had a chief complaint of a parkinsonian symptom. These records included data for 315 patients aged 40 years old or above, and 156 patients with at least one EEG record, with a total of 172 retrievable EEG records. After a thorough visual inspection, 14 EEG records were eliminated because of poor quality (i.e., presence of artifacts). A total of 158 EEG records belonging to 142 patients were analyzed and reviewed. Demographic and clinical information were retrieved for all 142 patients, including the final diagnosis and any cognitive scores obtained within 60 days of EEG recording. Thus, a total of 76 patients with 76 EEG records and a final diagnosis of IPD, CBD, MSA, or PSP were included in the analysis. There were a total of 19 IPD patients, 26 CBD patients, 13 MSA patients, and 18 PSP patients. The mean age of CBD and PSP patients were significantly higher than that of IPD patients, while that of MSA was the lowest. The proportions of female patients did not differ significantly between the four4 groups (Table 2).

**Table 2:** Demographic characteristics of patients and their cognitive scores.

	**IPD**	**CBD**	**MSA**	**PSP**	***p*-value**	***p*-value (CBD, MSA, and PSP, excluding IPD)**
Age	63.7 ± 11.1 (*n* = 19)	70.6 ± 9.6 (*n* = 26)	58.2 ± 10.4 (*n* = 13)	69.9 ± 5.8 (*n* = 18)	0.001	0.001
% Females	68.4%	57.7%	46.2%	61.1%	NS	
MMSE	28.6 ± 2.7 (*n* = 14)	25.8 ± 2.9 (*n* = 22)	27.5 ± 3.3 (*n* = 6)	24.3 ± 3.0 (*n* = 13)	0.003	NS
FAB	16.0 ± 2.2 (*n* = 5)	11.5 ± 3.75 (*n* = 9)	7.0 (*n* = 1)	10.3 ± 4.2 (*n* = 9)	0.044	NS

*Values are expressed as mean ± SD*.

*MMSE, Mini-Mental Status Examination; FAB, Frontal Assessment Battery; IPD, idiopathic Parkinson's disease; CBD, corticobasal degeneration; MSA, multiple systems atrophy; PSP, progressive supranuclear palsy; NS, not significant*.

MMSE scores were available for 14 IPD patients, 22 CBD patients, 6 MSA patients, and 13 PSP patients (Table 2). However, FAB was only available for a limited number of patients. IPD patients had higher mean cognitive scores, in terms of MMSE and FAB (Table 2) compared with the other three groups. On the contrary, the three groups of APDs (CBD, MSA, and PSP) had comparable MMSE and FAB.

### Major EEG Findings and GTE Score

**Figure 1:**
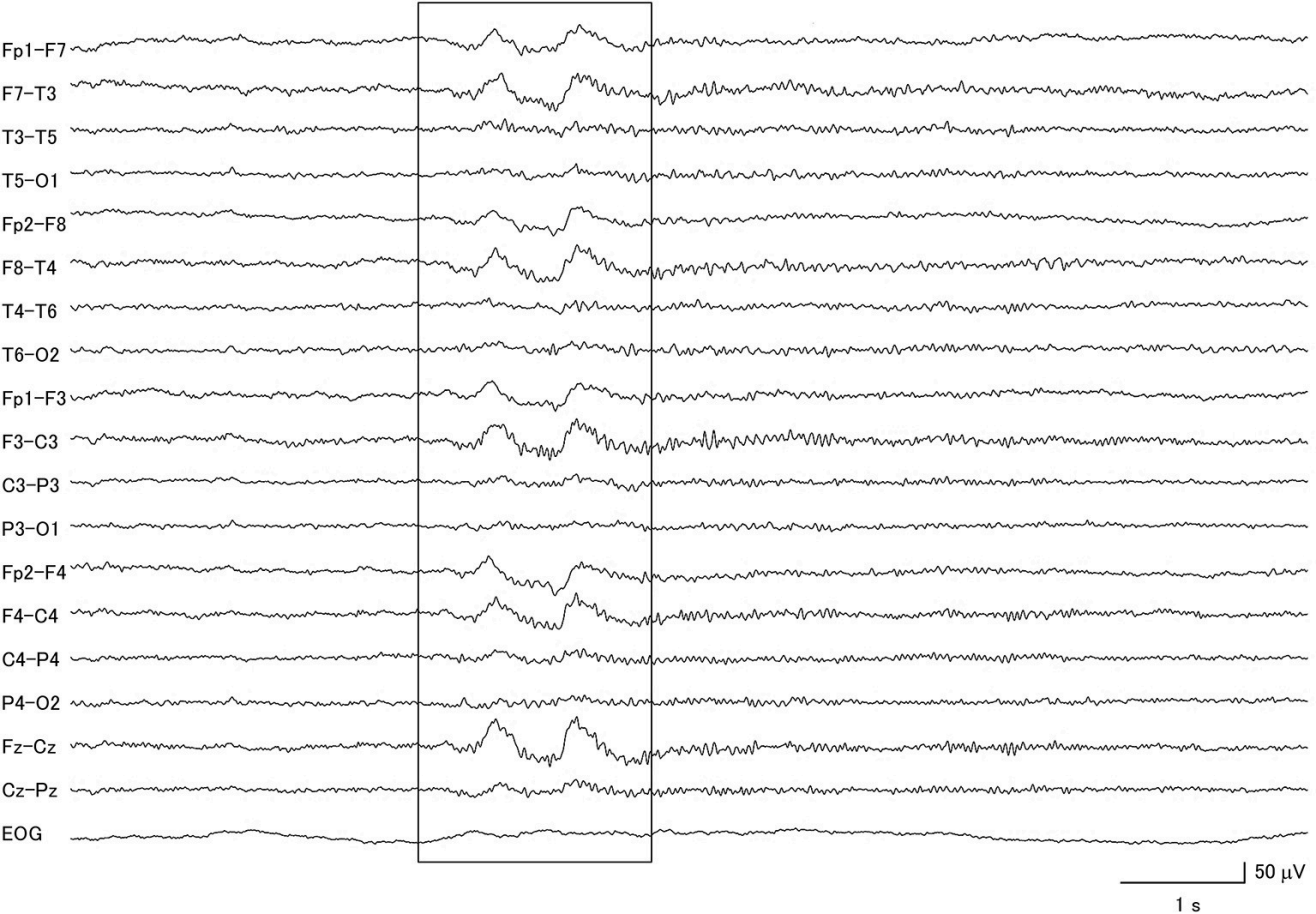
Electroencephalogram of a patient with idiopathic Parkinson's disease, demonstrating generalized delta slow wave activity (within box).

**Figure 2:**
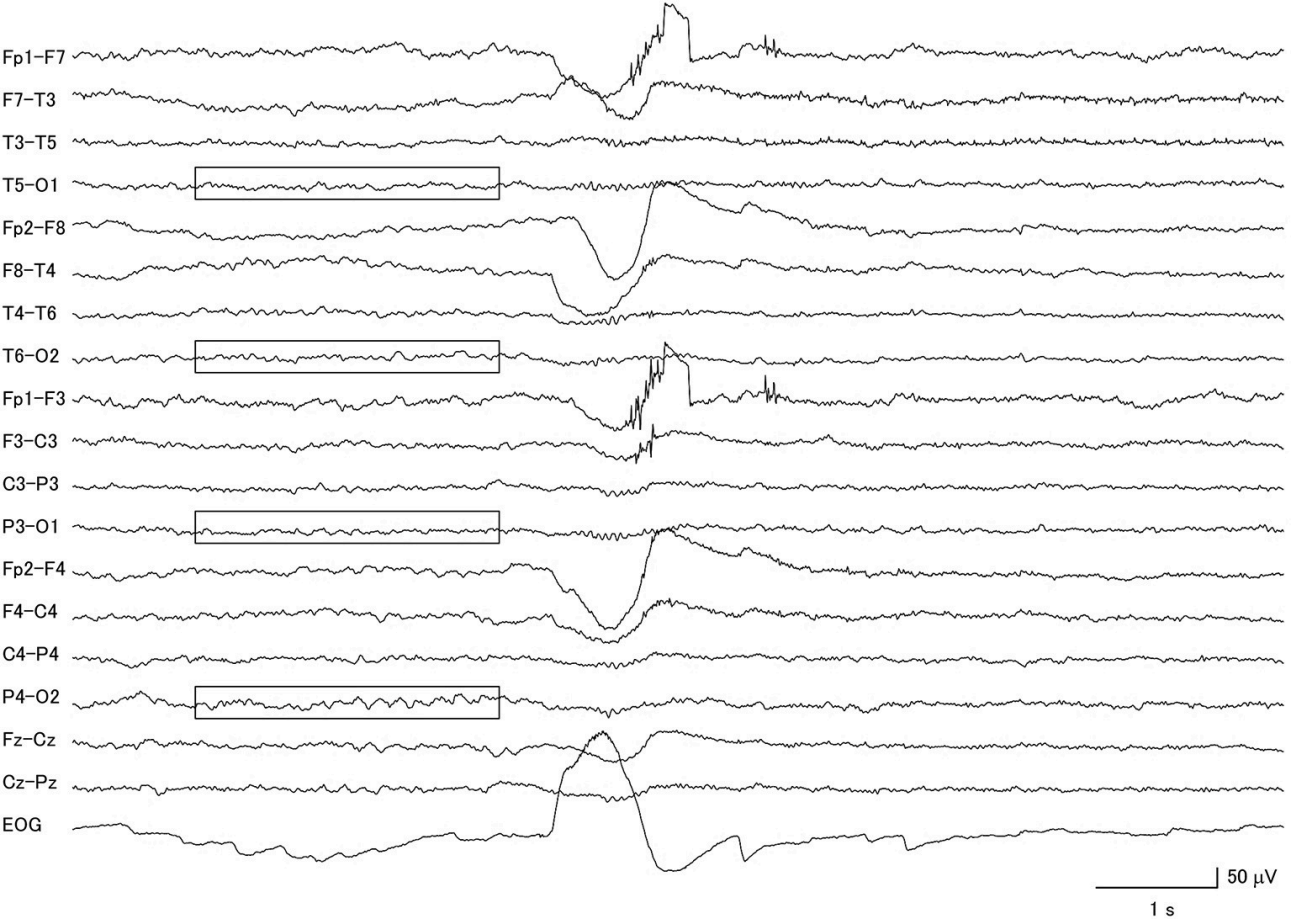
Electroencephalogram of a patient with corticobasal degeneration, demonstrating very low voltage patterns (within boxes). Notice that the background activity is also asymmetric.

**Figure 3:**
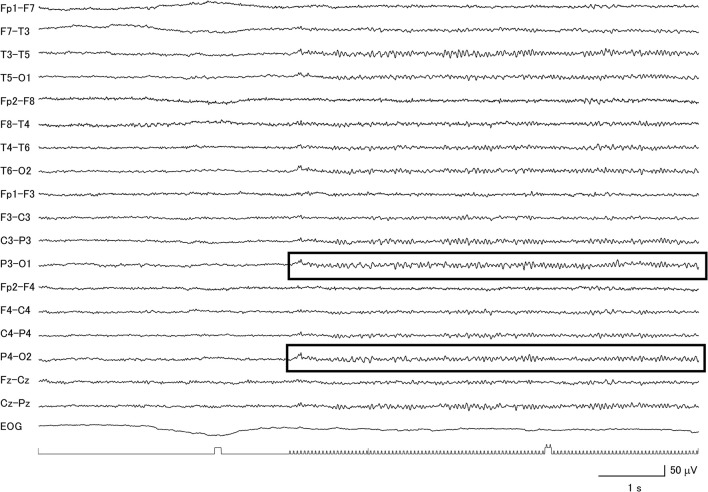
Clear evidence for a diffuse photic driving response (within boxes) in a patient with corticobasal degeneration with low voltage background activity.

**Figure 4:**
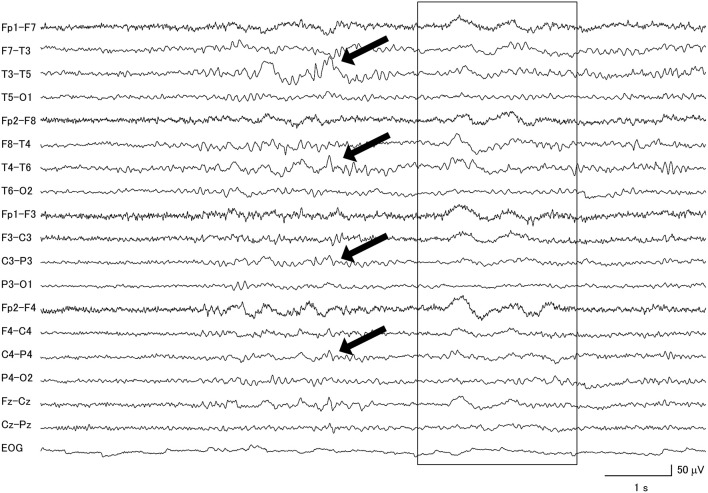
Focal slow wave activities (black arrows) were seen in the frontotemporal regions of a patient with progressive supranuclear palsy, combined with diffuse slow wave activity (within box).

In general, abnormal background activities, diffuse slow waves, and focal slow waves were all commonly found (Figures 1–4), being observed in 67.1, 65.8, and 57.9% of the patients, respectively. Among the patients with focal slow wave activities, 81.8% of these were either unilaterally located on the left side or left side-predominant, if bilateral. None of the 76 patients demonstrated epileptic focal abnormalities. IPD patients consistently scored the lowest for the total GTE score and in all subcomponents of the score, except for the diffuse slow wave activity subcomponent, in which MSA patients scored the lowest. CBD, MSA, and PSP patients had the highest mean scores in the rhythmic background activity, focal slow wave activity, and diffuse slow wave activity subcomponents, respectively (Table 3).

**Table 3:** Grand total EEG score in PD, CBD, MSA, and PSP patient groups.

	**IPD**	**CBD**	**MSA**	**PSP**	***p*-value**
*N*	19	26	13	18	
Grand total EEG score (median)	4.61 ± 2.88 (4.00)	5.96 ± 4.72 (6.00)	5.96 ± 3.72 (6.25)	6.64 ± 5.54 (6.00)	NS
Rhythmic background activity (median)	0.95 ± 1.08 (1.0)	1.42 ± 1.58 (1.0)	1.00 ± 0.91 (1.0)	1.22 ± 1.33 (1.0)	NS
Poor reactivity *n* (%)	2 (10.5%)	7 (26.9%)	1 (7.7%)	6 (33.3%)	**0.027**
Asymmetry *n* (%)	0	2 (7.7%)	0	0	NS
Very low voltage pattern *n* (%)	0	7 (26.9%)	0	0	**0.002**
Diffuse slow wave activity (median)	0.53 ± 1.07 (0)	1.12 ± 2.41 (0)	0.31 ±1.11 (0)	1.56 ± 2.73 (0)	NS
Focal slow wave activity (median)	3.13 ± 3.00 (3.0)	3.42 ± 3.78 (1.5)	4.65 ± 3.13 (4.5)	3.69 ± 3.66 (3.5)	NS
EOG abnormality *n* (%)	0	2 (7.7%)	1 (7.7%)	4 (22.2%)	**0.020**
GTE above 9	0	7 (26.9%)	4 (30.8%)	6 (33.3%)	**0.007**

*Values are expressed as mean ± SD, unless otherwise stated*.

*NS, not significant; IPD, idiopathic Parkinson's disease; CBD, corticobasal degeneration; MSA, multiple systems atrophy; PSP, progressive supranuclear palsy; EOG, electrooculogram; GTE, grand total EEG. Statistically significant values are in bold*.

Eight of the IPD patients were at stage 2–3 of the Hoehn and Yahr scale during the EEG recording (26), one was at stage 1, one was at stage 4, and 9 patients had no information available. In this group of patients, 57.9% had abnormal background activity, 21.7% had diffuse slow wave activity (Figure 1), and 57.9% had focal slow wave activity. None of these patients exhibited macro-square-wave jerks detected by EOG. The maximum GTE score among the IPD patients was 9.

Among the 26 CBD patients, 69.2% had abnormal background activity, 23.1% had diffuse slow wave activity, and 50.0% had focal slowing. Two patients had macro-square-wave jerks. Unique features in this group of patients were the presence of asymmetric background activity (7.7%) and a very low voltage pattern (26.9%) (Figure 2). Furthermore, among the 7 patients with the very low voltage pattern, 5 patients demonstrated a clear diffuse photoresponsiveness to intermittent photic stimulation (Figure 3). The maximum GTE score among the CBD patients was 17.5.

Abnormal background activity was seen in 61.5% of MSA patients and 76.9% had focal slow wave activity. This group had the highest mean score for the focal slow wave subcomponent, reflecting how commonly it was found in these patients. However, most of these focal slow wave activities were rare to occasional in terms of prevalence. Diffuse slow activity was an infrequent finding in this subgroup of APD, as it was only seen in 1 patient. The maximum GTE score among the MSA patients was 10.5.

Among the PSP patients, 77.8% had abnormal background activity, 27.8% had diffuse slow wave activity and 55.6% had focal slow wave activity (Figure 4). A patient from this group attained the highest GTE score in this study, which was 19.5.

Although the mean GTE scores across the four groups of patients did not significantly differ, analysis of the GTE score subcomponents showed significant differences in terms of scores for poor background reactivity and the presence of very low voltage patterns (Table 3). The CBD and IPD groups contained significantly higher proportions of patients showing poor background reactivity (26.9 and 33.3%, respectively). As mentioned earlier, the very low voltage pattern was only observed in CBD patients.

**Figure 5:**
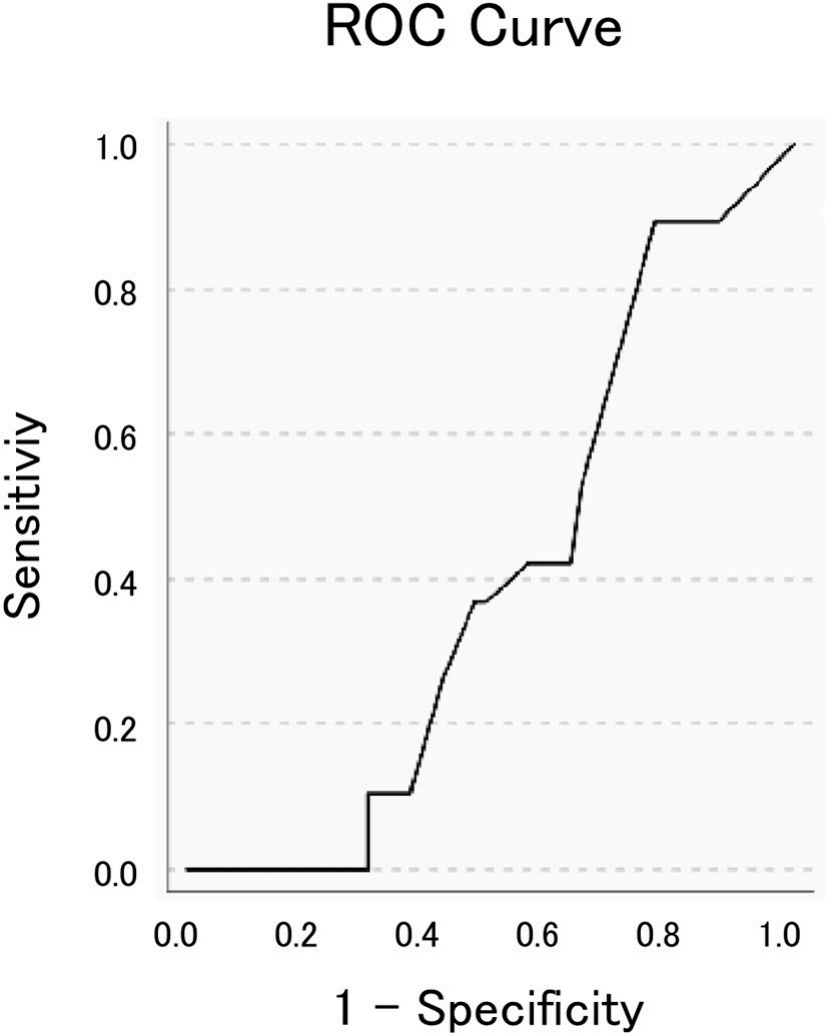
The receiver operating characteristic (ROC) curve fit to distinguish idiopathic Parkinson's disease from atypical parkinsonian disorders. The area under the curve was 0.589 (p = 0.236).

The ROC curve analysis (Figure 5), which aimed to distinguish IPD from APDs using the GTE score as the main variable, did not reach statistical significance (AUC = 0.589, *p* = 0.236). Nonetheless, it showed an optimum combination of sensitivity (89.5–100%) and specificity (29.8–33.3%) at a cut-off point of 9 or 8. Therefore, further analyses were performed using these values, showing a significant effect for a GTE score cut-off of 9 (*p* = 0.008), such that GTE scores of 9 and below had 100% sensitivity and 33.3% specificity in terms of distinguishing IPD from CBD, MSA, and PSP. To assess whether this result was influenced by the presence of confounders such as cognitive function or age, a covariance analysis was conducted. This revealed no significant interactions of these factors with the GTE score (*p* = 0.637).

### Unexpected EOG Findings

**Figure 6:**
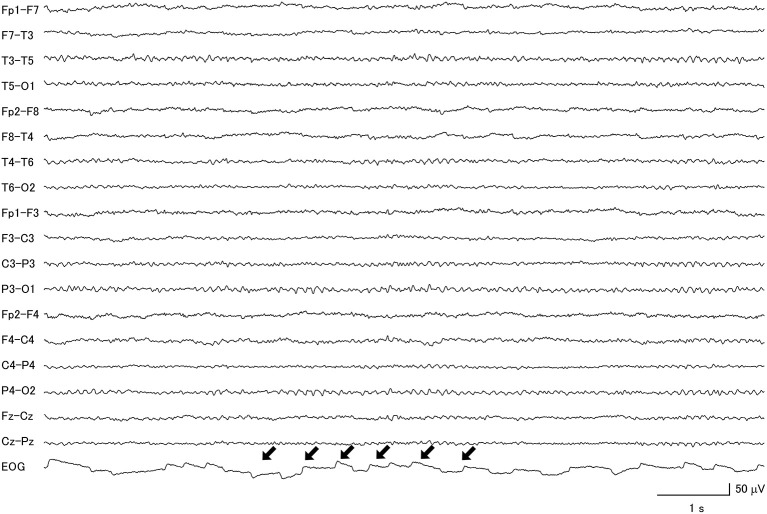
Macro-square-wave jerks (black arrows) were detected on the electro-oculogram of a patient with progressive supranuclear palsy.

Interestingly, the proportions of macro-square-wave jerks observed on the EOG channel (Figure 6) were significantly different between the four groups. They were observed in 1 (7.7%) MSA patient, 2 (7.7%) CBD patients, 4 (22.2%) PSP patients, and none (0%) of the IPD patients (Table 3).

## Discussion

The ongoing revolution in neurodegenerative disease research has led to novel findings relating to symptomatology, neuroimaging, genetic markers, molecular biology, targeted therapies, and disease models. It has made it possible to differentiate IPD from APD and even to diversify the once “homogenous” subsets of APDs into several clinical subvariants (15–17). However, lack of access to these tests in different countries, especially in countries in the developing world, currently limits their use.

In this study, we investigated whether a simple semi-quantitative EEG assessment tool, namely the GTE scoring system, is useful in differentiating IPD from APD, as well as distinguishing different APD subvariants from each other.

From the retrospective review of patient EEG data, we were also able to present a detailed description of EEG findings from a group of parkinsonian disorders seen at Kyushu University Hospital, a tertiary hospital.

The ages of the populations in this study are in line with data from the literature, in that CBD and PSP patients are on average afflicted with the disease during their 6–7th decade of life, while MSA patients begin to experience the disease at a relatively younger age of 55–60 years old (15, 27). IPD afflicts a wide age range and the prevalence increases dramatically over the age of 40 years (27, 28).

It is evident that IPD patients have the fewest EEG abnormalities as measured by GTE score. This is consistent with findings in the literature, which state that IPD patients without dementia tend to have relatively preserved EEG activity (29). Only 2 patients exhibited poor background reactivity, which is also in line with the literature because this particular finding is more associated with Parkinson's disease with dementia (30, 31). Because the sample population of IPD patients in this study had preserved cognitive function, the finding of slowed background activities may be attributed to motor disability (30). Unfortunately, a lack of available data for the Hoehn and Yahr stage of 9 patients prevented us from analyzing the relationship of GTE score to this diagnostic measurement. Hence, such a claim cannot currently be backed up with sufficient statistical evidence.

Previous studies of CBD patients have already reported finding asymmetric background EEG activity (32, 33). Such activity was observed in 7.7% of our CBD patients, which is comparable to the value of 6.7% from a previous report (33). Notably, the striking finding of low voltage background activity in this study, defined as <20 μV on a bipolar montage (34), has never been mentioned in the literature. This pattern is commonly seen in other neurodegenerative disorders, such as Huntington's disease (35), and in the later stages of Creutzfeldt-Jakob disease (36). Thus, it can be explained as a consequence of these kinds of progressive neurodegeneration. Moreover, the diffuse photoresponsiveness observed in the majority of these patients might be explained either by a state of hyper-excitability or by a state involving dampened inhibitory responses. In the context of neurodegeneration, perhaps the latter is a more plausible mechanism. Diffuse slow wave and focal slow wave activities are common findings in EEG data from CBD patients (28), as was also evident in our study.

EEG findings from MSA patients have been reported to be normal (28). This is inconsistent with our findings, which showed that a high proportion of MSA patients showed abnormal EEG activity. In this APD subgroup, the combination of abundant focal slow wave activities and the rare occurrence of diffuse slowing is a characteristic finding, but one that most books or journals have not yet pointed out. Although these findings are considered to be non-specific, our results suggest that for MSA patients with abnormal EEG, focal slowing is frequently encountered, while diffuse slowing is rare.

EEG traces from PSP patients have been reported to show non-specific abnormalities (37), which is not contradictory with the results of our study. However, previous reports of occasional epileptic discharges (38), frontal intermittent rhythmic delta activity (FIRDA) (32), and the presence of low voltage patterns (32) were not replicated in our study. Nevertheless, this subgroup of APD patients had the highest diffuse slow wave activity among the three subgroups.

In the group of patients with parkinsonian disorders studied here, non-specific abnormalities such as abnormal background activity, diffuse slow waves, and focal slow waves were all very common findings. We noted that focal slow activities were most common in the left hemisphere, especially in frontotemporal regions, an observation has also been noted in earlier studies (39, 40). Such peculiar findings have been hypothesized to be attributed to cytoarchitectural differences between the two hemispheres, vulnerability of the left hemisphere to injury, left-sided predisposition to vascular disturbances or to the finding that the greatest cortical cell loss occurs in the superior temporal gyrus in neurodegenerative diseases (39, 40). Neuromodulators concentrations have been shown to correlate strongly with EEG frequency patterns. The monoamine-acetylcholine (ACh) balance hypothesis states that slow rhythms, particularly at delta frequency, are a reflection of monoaminergic receptor effects (41). Indeed, it is well-known that widespread alterations in dopamine levels occur in parkinsonian disorders. Norepinephrine and serotonin levels are also altered in these diseases (28). These monoaminergic system alterations contribute to the slow EEG activities documented for all four of the parkinsonian disorders examined here. The monoamine-ACh balance hypothesis also states that alpha rhythm is a reflection of the cholinergic system (41). IPD patients have variable cholinergic denervation, with normal neocortical and thalamic ACh activity in 64% of patients (42). Choline acetyltransferase (ChAT) activity is also variable in MSA patients, with activity levels either low or normal (28). Although CBD and PSP patients both show decreased ChAT activity, this occurs in different regions of the brain. CBD patients show reduced ChAT activity in the paracentral region, and in frontal, parietal and occipital cortices, while PSP patients show reductions in the paracentral region and thalamus (43). Our study showed a relatively preserved alpha background activity in IPD and MSA patients compared with CBD and PSP patients, which is consistent with the monoamine-ACh hypothesis.

It is also interesting to note that macro-square-wave jerks on the EOG trace were observed in patients with APD but not IPD. This was also reported in a previous study, in which macro-square-wave jerks of more than 1° in amplitude were frequently seen in PSP and MSA patients, but not in IPD patients (44). Furthermore, a review paper on the topic did not find that macro-square-wave jerks were a consistent finding in CBD (45).

In summary, this study yielded results that differed from our original hypothesis, which predicted that a modified GTE score would be able to distinguish subgroups of APD. Nevertheless, even though the ROC curve analysis could not distinguish IPD from APD at a statistically significant level, chi-square analysis using a GTE score of 9 did permit a statistically significant discrimination of IPD from APD. At this cut-off value, this method has excellent sensitivity (100%), but poor specificity (33.3%).

There were several limitations for this research, including: the lack of histopathologic diagnosis; incomplete neuroimaging studies, missing clinical information on several patients (i.e., cognitive scores, Hoeh, and Yahr scale); the fact that the duration of illness and current medications were unaccounted for; and the unmatched age and cognitive functional status of the population groups. We attempted to increase the sample size in this study by extending the period from which records were included to 12 years. By addressing these limitations and further revising our modified GTE score, semi-quantitative EEG analysis could be further explored to clarify its diagnostic potential for parkinsonian disorders.

## Conclusion

EEG is a non-invasive measurement of brain function for which semi-quantitative analysis can easily be performed. Our adopted modified GTE score was able to distinguish IPDs from CBD, PSP or MSA at a cut-off score of 9 with excellent sensitivity but poor specificity. However, it lacked diagnostic power in differentiating a particular subvariant of APD from other APDs. Future studies should investigate semi-quantitative EEG analysis to explore its diagnostic potential for parkinsonian disorders.

## Ethics Statement

This study was carried out in accordance with the recommendations of Ethics Committee at the Graduate School of Medical Sciences, Kyushu University. Informed consent was waived for the following reasons: (1) the study involved retrospective review of data, (2) the study posed no threat nor harm to the human subjects, (3) the study did not involve use of private information of the subjects.

## Author Contributions

EB: data collection, statistical analysis, and writing. TM and JY: data collection. TU and KO: data collection, and statistical analysis. JK: writing. ST: data collection, study design, statistical analysis, and writing.

### Conflict of Interest Statement

The authors declare that the research was conducted in the absence of any commercial or financial relationships that could be construed as a potential conflict of interest.
